# Yeast Secretes High Amounts of Human Calreticulin without Cellular Stress

**DOI:** 10.3390/cimb44050122

**Published:** 2022-04-19

**Authors:** Rūta Zinkevičiūtė, Raimundas Ražanskas, Algirdas Kaupinis, Neringa Macijauskaitė, Evaldas Čiplys, Gunnar Houen, Rimantas Slibinskas

**Affiliations:** 1 Department of Eukaryote Gene Engineering, Institute of Biotechnology, Life Sciences Center, Vilnius University, Saulėtekio av. 7, LT-10257 Vilnius, Lithuania; raimundas.razanskas@bti.vu.lt (R.R.); neringa.macijauskaite@bti.vu.lt (N.M.); evaldas.ciplys@bti.vu.lt (E.Č.); rimantas.slibinskas@bti.vu.lt (R.S.); 2 Proteomics Centre, Institute of Biochemistry, Life Sciences Center, Vilnius University, Saulėtekio av. 7, LT-10257 Vilnius, Lithuania; algirdas.kaupinis@gf.vu.lt; 3 Department of Biochemistry and Molecular Biology, University of Southern Denmark, Campusvej 55, DK-5230 Odense, Denmark; gunnarh@bmb.sdu.dk

**Keywords:** calreticulin, secretion, yeast, 2DE, LC-MS^E^, proteomics, recombinant protein, cellular stress

## Abstract

The ER chaperone calreticulin (CALR) also has extracellular functions and can exit the mammalian cell in response to various factors, although the mechanism by which this takes place is unknown. The yeast *Saccharomyces cerevisiae* efficiently secretes human CALR, and the analysis of this process in yeast could help to clarify how it gets out of eukaryotic cells. We have achieved a secretion titer of about 140 mg/L CALR in our *S. cerevisiae* system. Here, we present a comparative quantitative whole proteome study in CALR-secreting yeast using non-equilibrium pH gradient electrophoresis (NEPHGE)-based two-dimensional gel electrophoresis (2DE) as well as liquid chromatography mass spectrometry in data-independent analysis mode (LC-MS^E^). A reconstructed carrier ampholyte (CA) composition of NEPHGE-based first-dimension separation for 2DE could be used instead of formerly commercially available gels. Using LC-MS^E^, we identified 1574 proteins, 20 of which exhibited differential expression. The largest group of differentially expressed proteins were structural ribosomal proteins involved in translation. Interestingly, we did not find any signs of cellular stress which is usually observed in recombinant protein-producing yeast, and we did not identify any secretory pathway proteins that exhibited changes in expression. Taken together, high-level secretion of human recombinant CALR protein in *S. cerevisiae* does not induce cellular stress and does not burden the cellular secretory machinery. There are only small changes in the cellular proteome of yeast secreting CALR at a high level.

## 1. Introduction

Calreticulin (CALR) is a protein best known for its role in the endoplasmic reticulum (ER)—where it acts as a chaperone in protein quality control [1], regulates Ca^2+^ homeostasis and Ca^2+^-dependent pathways [2], and is involved in MHC class I antigen processing [3]. It has been well established, that calreticulin can leave the ER and be localized in the nucleus, cytosol, on the cell surface or in the extracellular compartments [4]. There it has important functions in cellular proliferation [5], cell adhesion [6,7], cell migration [8] and phagocytosis of apoptotic cells [9]. Furthermore, it plays a role in the adaptive immune response [10], in the uptake of CALR-expressing cancer cells by dendritic cells [10] and in wound healing [5,11]. Despite the cell surface-bound and exogenous CALR involvement in a wide variety of cellular functions, the way CALR gets out of the cell remains unclear [12]. It has been shown that in some cell types, the exit can be induced by ER stress, which can be provoked by various stimuli such as anthracyclines [13], reduction in ER Ca^2+^ levels [14], hypoxia, high temperature or pH imbalance [4] or loss of the C-terminal KDEL motif due to mutations in the CALR gene [15]. Since CALR possesses an ER-retrieval KDEL amino acid sequence, it can travel from the ER to the Golgi complex but will always be retrieved to the ER, and as it is a non-glycosylated protein, it lacks the usual requirements for proteins to be exposed via anterograde secretory pathways [16]. Surprisingly, active, high-level secretion of mature native recombinant CALR protein was reported in yeast [17]. It is possible that analysis of this efficient and non-detrimental secretion of CALR may hold some answers to how CALR exits human cells.

The aim of this study was to perform a comparative whole proteome analysis of CALR-secreting yeast *Saccharomyces cerevisiae* cells versus control cells harbouring an empty vector, using non-equilibrium pH gradient electrophoresis (NEPHGE)-based two-dimensional electrophoresis (2DE) and liquid chromatography-mass spectrometry in data-independent analysis mode (LC-MS^E^) to try to identify cellular proteins that are likely to participate in the CALR secretion pathway. We chose to use NEPHGE-based 2DE, although it is more labour-intensive and less broadly used than immobilized pH gradient (IPG) based 2DE, is still instrumental in more specialized laboratories. The capability of NEPHGE-based 2DE to better resolve basic protein spots in comparison with IPG [18] is exploited for functional proteomics experiments in a broad pH range [19], as well as for experiments with highly basic proteins [20,21]. While an improved protocol on NEPHGE-based 2DE was published in 1995 by Klose and Kobalz [22], it was commercialized only later by WITA GmbH [23] and the “WITAvision” 2DE system was developed. Unfortunately, this company ceased operations and left owners of the apparatus without a supply of reagents. The composition of the CA in isoelectric focusing (IEF) gels sold by WITA GmbH still remains unknown and recreating the original CA mixture presented by Klose and Kobalz, also posed a challenge, due to the discontinuation of the production of most of the ampholytes used at that time. Despite this, we here show a restored CA composition for NEPHGE-based 2DE, which shows a high positive correlation to formerly commercially available gels.

In this comparative proteomics experiment between CALR-secreting and control cells with an empty vector, we quantified protein abundance from 2DE gels made with our restored CA mixture, and also, we quantified all peptides present in the whole proteome samples analysed by LC-MS^E^, using label-free quantitation via TOP3-approach. We found that effective secretion of CALR does not induce cellular stress or the expression of any secretory pathway proteins and only results in a slightly higher demand for energy and nutrients in *S. cerevisiae*.

## 2. Materials and Methods

### 2.1. Plasmids, Yeast Strains, Media and Growth

In this study, we used two plasmids (See Figure S5A,B):

pFGAL7-CRT—for inducible expression and secretion of human CALR protein. pFGAL7 vector was derived from pFGG3 vector [23] by removing GAL10/PYK1 promoter using SmaI (Thermo Scientific, Vilnius, Lithuania) and XbaI (Thermo Scientific, Vilnius, Lithuania) restriction endonucleases (RE). This allows stronger expression of genes under the sole GAL7 promoter. A gene encoding full-length wild-type human CALR precursor (GenBank Acc. no. M84739 for cDNA sequence and UniProtKB acc. No. P27797 for amino acid sequence) was synthesized by GenScript (Piscataway, NJ, USA). The gene was subcloned into the yeast expression vector pFGAL7 under control of galactose-inducible yeast *GAL7* gene promoter using BcuI (Thermo Scientific, Vilnius, Lithuania) RE. DNA manipulations were conducted according to standard procedures [24]. *CALR* gene sequence in pFGAL7-CRT vector was verified by Sanger sequencing;

pFGG3—as an empty control vector (generation of which is described in [23]);

The aforementioned plasmids were used to transform *S. cerevisiae* strains AH22 (MATa leu2-3 leu2-112 his4-519 can1 [KIL-o]), BY4741 (MATa his3Δ1 leu2Δ0 met15Δ0 ura3Δ0), BY4741 ΔSOD1 (MATa his3Δ1 leu2Δ0 met15Δ0 ura3Δ0 ΔSOD1) (yeast knockout collection) using the conventional LiCl method [24];

*S. cerevisiae* cell cultures were grown in YEPD medium (yeast extract 1%, peptone 2%, glucose 2%) with 5 mM of formaldehyde added. CALR was then expressed in an induction medium YEPG (yeast extract 1%, peptone 2%, galactose 2.5%). Shortly, *S. cerevisiae* cells transformed with plasmid carrying human *CALR* gene or an empty control vector were inoculated into YEPD medium with 5 mM of formaldehyde, grown overnight and then re-inoculated into fresh YEPD medium with 5 mM of formaldehyde to 0.05 OD_600_. The cell culture was then grown for 21 h, centrifugated for 5 min at 800× *g* room temperature, the old growth media discarded, fresh induction medium YEPG with 5 mM of formaldehyde added, and the cells resuspended. After change of the growth medium, cells were grown for 18–21 h and then harvested by centrifugation and stored at −70 °C. The culture growth medium was harvested by pelleting a portion of the cell culture and collecting the medium at different time points ranging from 3 to 18 h after the induction of CALR expression with YEPG medium.

### 2.2. Cell Culture Medium Sample Preparation and SDS-PAGE

Freshly harvested culture medium was mixed in equal parts with 2× SDS-PAGE sample buffer (125 mM Tris-HCl, pH 6.8, 20% glycerol, 8% sodium dodecyl sulphate (SDS), 150 mM of dithiothreitol (DTT), 0.01% bromophenol blue) and immediately boiled for 8 min. Prepared media samples were loaded onto an SDS-PAGE gel, 16 µL per well, and the electrophoresis was run in SDS-Tris-glycine buffer. PAA gels were fixed for 15 min in fixation solution (50% ethanol, 40% HPLC grade water, 10% acetic acid) and stained for 25 min with Coomassie Brilliant Blue R-250 (50% ethanol, 10% acetic acid, 0.1% Coomassie Brilliant Blue R-250 (CBB R-250), 40% HPLC grade water) followed by destaining in 5% acetic acid. Purified CALR protein (UAB Baltymas) used for densitometric quantification was prepared correspondingly but diluted with 1× SDS-PAGE sample buffer to load different concentrations onto the PAA gel.

### 2.3. Cell Sample Preparation for 2DE

A total of 300–500 mg of cell pellets were collected into a glass test-tube by centrifugation, washed with distilled water and frozen at −80 °C. After storing, the cells were placed on ice and resuspended in 3 volumes (*v*/*w*) of denaturing IEF buffer containing 7 M urea, 2 M thiourea, 2% CHAPS detergent, 1% ampholytes (pH 3–10, Pharmalyte, GE Healthcare, Chicago, IL, USA), 0.002% Bromophenol Blue and 75 mM DTT (added just before use). The cells were lysed by adding a glass bead volume twice the weight of cell pellets and vortexing at high speed 8 times for 30 sec. Samples were cooled for 10 sec on ice following 30 sec at room temperature between each vortexing. Cell debris was removed by centrifugation at 16,000× *g*, for 15 min at 16 °C. Cell lysate supernatants were collected, and protein concentrations were measured using a modified Bradford’s protein assay (Roti-Nanoquant, Carl Roth GmbH, Baden-Wurttemberg, Germany). Samples were diluted with IEF buffer to equalize the protein concentrations. Prepared yeast cell lysates were stored frozen at −80 °C.

### 2.4. Making of NEPHGE Gels

Non-equilibrium pH gradient electrophoresis (NEPHGE) gels were made with a non-linear pH gradient formed by carrier ampholytes (CA). Each CA mixture was designed with narrow and broad pH range ampholytes commercially available at the time. Our restored “New mix” ampholyte blend (Mix no. 3 in the Table S1) consists of: Servalyt pH 2–11 (Serva Electrophoresis GmbH, Heidelberg, Germany)—1 part; Pharmalyte pH 5–8 (SigmaAldrich, St. Louis, MO, USA)—2 parts; Pharmalyte pH 4–6,5 (SigmaAldrich, 5--8 (SigmaAldrich, St. Louis, MO, USA)—3 parts; Ampholyte high-resolution pH 6–9 (Carl Roth)—1 part; Ampholyte high-resolution pH 3–10 (Carl Roth, Baden-Wurttemberg, Germany)—1 part; total of 8 parts. This CA mixture was then incorporated into two essential NEPHGE gel solutions—separation gel (Sep gel) and capping gel (Cap gel) described in [22].

### 2.5. Casting NEPHGE First-Dimension Gels for 2DE

Shortly, Sep gel and Cap gel were cast in succession in a vertical device for casting two-layered rod gels for the first dimension. The Sep gel takes up about 2/3 and the Cap gel 1/10 to 1/20 of the glass rod mold–the rest is left for the sample. For the 8 cm length isoelectric focusing (IEF) gel (11 cm rod mold), 500 µL of Sep gel with 12 µL 0.8% ammoniumpersulfate (APS) and 100 µL of Cap gel with 2.5 µL of 0.8% APS is required (all solutions are degassed by sonication). After casting, the initial polymerization of the Cap gel was achieved by leaving the gel undisturbed for 30 min at room temperature. Later, the rod mold was removed from the casting device, a drop of distilled water was placed on top of the rod on the sample loading side (to prevent drying out), ends of the rod were covered with caps or parafilm wax and left to fully polymerize for 72 h. WITA NEPHGE gels were cast almost identically using a set of standardized materials.

### 2.6. Running the First Dimension

After the full polymerization of rod gels, the first-dimension separation of proteins was performed in a vertical electrophoresis device [22]. The lower chamber of the unit was filled with 400 mL of degassed cathode buffer (20 g of glycerol, 216 g of urea, and 170 mL aqua dist., filled up to 380 mL; then 20 mL of ethylenediamine added; solution prepared on a 40 °C heating plate). The rod gels were fixed in the device with their ends submerged into the cathode buffer and the water cork was removed. Sample solutions containing 80–200 µg of proteins from whole cell lysates were mixed with pre-heated (to 50 °C) agarose-supplemented ampholyte phosphate buffer in a ratio of 4:1, immediately applied to the anode ends of the capillary gels leaving no trapped air bubbles, covered with 10 µL of sample-stabilizing overlay solution [25] and left to set for 5–10 min. After the solidification of a sample, 400 mL of degassed anode buffer (72 g of urea, and 300 mL aqua dist., filled up to 380 mL; with the addition of 20 mL of phosphoric acid) was poured into the upper chamber submerging the rod gels. The first-dimension electrophoretic separation of proteins was conducted by using the following sequence of running conditions: for small 11 cm rods—100 V for 1 h 15 min; 200 V for 1 h 15 min; 400 V for 1 h 15 min; 600 V for 1 h 15 min; 800 V for 10 min; and 1000 V for 5 min. After the electrophoresis had ended, the rod gels were carefully pushed out of the glass tube molds onto plastic rails with the help of a syringe. Then, the gels were adapted to the conditions of the second dimension by a series of three 15 min incubations in equilibration buffer (125 mM of Tris-H_3_PO_4_ (125 mM trishydroxymethylaminomethane solution with the pH brought to 6.8 with phosphoric acid), 40% of glycerol, 3% of SDS) and 75 mM of DTT, following three 15 min equilibrations in the same buffer with 125 mM of 2-iodoacetamyde (IAC). The equilibrated gels were stored in −80 °C before the application to the second-dimension separation system.

### 2.7. Running the Second Dimension (SDS-PAGE), Fixing and Staining of 2DE Gels

SDS-PAGE running conditions, fixing and staining were performed identically as described earlier [18].

### 2.8. Image Analysis

All gels were scanned using ImageSanner III (GE Healthcare, Chicago, IL, USA) using blank filter, transparent mode and 300 dpi resolution. Densitometric analysis of 1D SDS-PAGE gels was performed with ImageQuant TL (GE Healthcare, Chicago, IL, USA) using default settings.

Images of 2DE gels cast with our “New mix” and “WITA” solutions were analysed using PDQuest 8.0.1 2D (BioRad, Hercules, CA, USA) analysis software by following the manufacturer‘s recommendations. The coefficient of correlation (r) was calculated by comparing each gel to another. Pearson‘s coefficient of correlation measures the linear association of two variables and ranges from −1 to +1, with r = 1 meaning a strong linear association [26].

2DE images of CALR-secreting yeast cell samples versus non-secreting controls were analysed using ImageMaster 2D Platinum 7.0 software (GE Healthcare, Chicago, IL, USA). Protein spots were detected automatically by setting the same parameters for all analysed 2D gels. Artefact spots and speckles (mostly near the boundaries of the gels) were deleted manually in every 2D gel. Then gels were matched in separate groups of three gels followed by matches between the groups according to required comparison. Differentially expressed cellular proteins between CALR-expressing cultures and control were evaluated by calculating the fold change (FC)—the ratio of %Vol between spots. Our selected threshold for FC was 1.5 times (0.58 log2FC)—protein spots that show a 1.5-fold increase or decrease in volume are thought to be differentially expressed. The t-test analysis of spot value ratio between CALR and control samples was used to distinguish the spots that were significantly differentially expressed. In parallel, we used DESeq2—a differential analysis of count data that uses shrinkage estimation for data dispersion and FC—to quantitatively asses the strength of differential expression in datasets with few replicas [27]. This analysis evaluates how significantly different the mean expression levels between sample groups are depending on the user-selected fc threshold (for calculations see Spreadsheet S1 in Supplementary Materials).

### 2.9. Sample Preparation for LC-MS^E^

Samples of proteins excised from gels were prepared as described by Shevchenko et al. [28]

Whole proteome samples were digested with trypsin according to FASP protocol as described by Wisniewski et al. [29] Briefly, samples were diluted in 8 M urea following two washes with urea and alkylated with 50 mM of IOA (GE Healthcare Life Sciences, Marlborough, MA, USA). Protein concentrators were washed twice with urea and twice with 50 mM of ammonium bicarbonate. Proteins were digested overnight with TPCK Trypsin 20233 (Thermo Scientific, Waltham, MA, USA). After overnight digestion, peptides were collected from the concentrators by centrifugation at 14,000× *g* for 10 min and additionally eluted using 20% acetonitrile. The eluates were mixed, acidified with 10% trifluoracetic acid and lyophilized in a vacuum centrifuge. The lyophilized peptides were redissolved in 0.1% formic acid.

### 2.10. LC-MSE (Data-Independent Acquisition)-Based Protein Identification

Liquid chromatography (LC) was performed using a Waters Acquity ultra-performance LC system (Waters Corporation, Wilmslow, UK). ACQUITY UPLC HSS T3 250 mm analytical column was used to perform peptide separation. Data was acquired using Synapt G2 mass spectrometer and Masslynx 4.1 software (Waters Corporation, Wilmslow, UK) in positive ion mode using data-independent acquisition (DIA) coupled with ion mobility separation (IMS, *UDMS*^E^) [30]. For the survey scan, the mass range was set to 50–2000 Da with a scan time set to 0.8 s. Raw data are available via the MassIVE repository with identifier MSV000088879. Raw data were lock mass-corrected using the doubly charged ion of [Glu1]-fibrinopeptide B (m/z 785.8426; [M+2H]2+) and a 0.25 Da tolerance window. The raw data were processed with the ProteinLynx Global SERVER (PLGS) version 3.0.1 (Waters Corporation) Apex3D and Pep3D algorithms to generate precursor mass lists and associated product ion mass lists for subsequent protein identification and quantification. Peak lists were generated using the following parameters: (i) low energy threshold was set to 150 counts, (ii) elevated energy threshold was set to 50 counts, and (iii) intensity threshold was set to 750 counts. Database searching was performed with PLGS search engine using automatic peptide tolerance and fragment tolerance, minimum fragment ion matches of 1 per peptide and 3 per protein, FDR < 4%. Trypsin as the cleavage protease was used for data analysis, one missed cleavage was allowed, and fixed modification was set to carbamidomethylation of cysteines, the variable modification was set to oxidation of methionine. UniprotKB/SwissProt *Saccharomyces cerevisiae* databases (24 September 2020) with bovine trypsin (TRY1_BOVIN) were used for protein identification. Label-free quantification using the TOP3-approach was used for the quantification of proteins. TOP3 intensity was calculated as the average intensity of the three best ionizing peptides using ISOQuant [31]. The maximum FDR of protein identification was set to 1%. Log2 transformation was applied to the data before computing fold-changes. The Bayes algorithm of the limma Bioconductor package was used to compute the log2 fold-changes and *p*-values. The calculated *p*-values are the adjusted FDRs using the Benjamini–Hochberg procedure.

### 2.11. Bioinformatic Analysis of Differentially Expressed Proteins

Functional enrichment analysis was performed by analysing differentially expressed proteins identified by LC-MS^E^, against yeast *S. cerevisiae* genome set of GO database [32] using R package clusterProfler 4.0 [33].

## 3. Results

### 3.1. Restoration of the NEPHGE First-Dimension Gel CA Composition and Comparison to the Commercial “WITA” Gels

With the aim to recreate a CA mixture forming a stable, non-linear pH 3–10 gradient for NEPHGE-based first-dimension separation, we tested 12 different CA compositions in NEPHGE gel solutions (See Table S1). The main constitution of CAs and other components of the gels were made according to an updated protocol by Klose and Kobalz from 1995, with slight variations between proportions, pH ranges and manufacturers. CAs of short pH range of 4–6.5 were at the highest concentration, followed by CAs pH 5–8 and pH 6–9 for increased separation capacity in the 2DE gel zone generally featuring the highest spot density. CAs of broad pH range (pH 2–11 and pH 3–10) were used to widen the pH gradient, and another narrow range CA pH 8–10.5 was added to increase the resolution at the alkaline end of the pH gradient, where proteins with basic pI tend to accumulate. After casting, the first-dimension gels were loaded with the same sample (80 μg of lysate of *S. cerevisiae* AH22 strain cells transformed with pFGG3 empty vector (for sample preparation see Section 2) followed by second-dimension separation. We analysed the protein patterns in all 12 gels (see Figure S1) searching for qualitative and quantitative differences, and we chose CA mix No. 3 (“New mix”) as the best performing one. Despite the fact that the ampholytes themselves differ, the composition of this mixture most closely resembles that described by Klose and Kobalz [22] (see Section 2).

**Figure 1:**
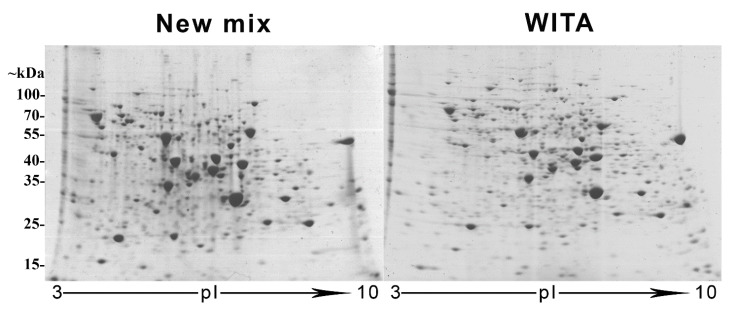
Two-dimensional electrophoresis (2DE) gels made using either our restored “New mix” or commercial “WITA” solutions for non-equilibrium pH gradient electrophoresis (NEPHGE)-based first-dimension isoelectric focusing (IEF). Both gels were loaded with the same sample (80 μg of lysate of *S. cerevisiae* AH22 strain cells transformed with pFGG3 vector) and second-dimension SDS-PAGE were performed identically.

**Figure 2:**
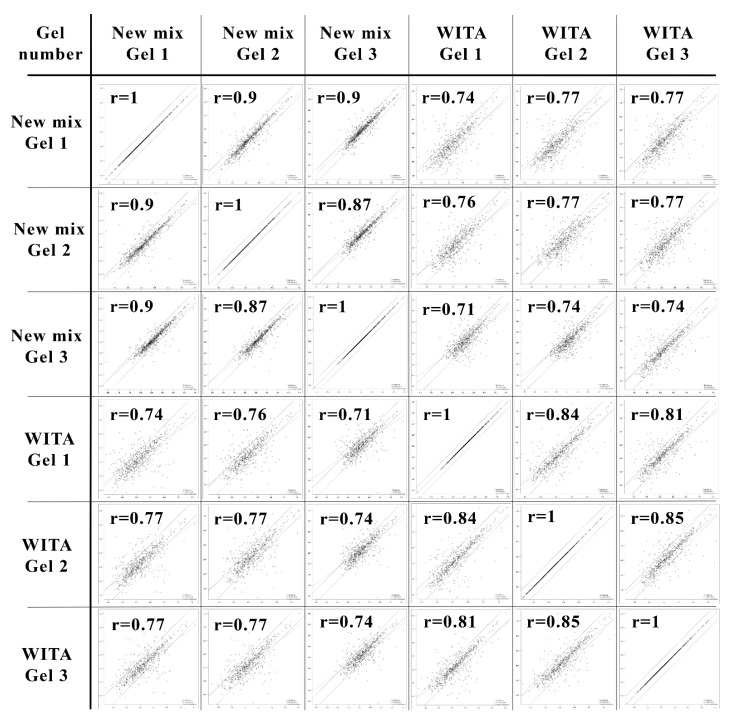
Scatter plot analysis and correlation coefficients (r) between 2DE gels made with the “New mix” and commercial “WITA” ampholyte mix. For the comparison, three runs of identical gels of each constitution (“New mix” or “WITA”) were compared with each other. The correlation coefficient r = 1 references the comparison of a gel with itself, which indicates strongest correlation possible. The closer r is to 1, the stronger it is.

Secondly, we compared 2DE gels made with our re-created “New mix” and the formerly commercially available “WITA” pre-made gel solutions. We loaded the gels with the same sample of crude lysate of *S. cerevisiae* strain AH22 cells transformed with an empty pFGG3 vector. The running conditions, second-dimension SDS-PAGE, gel fixing and staining with Coomassie R-250 were identical (See Section 2, Figure 1). We analysed the 2DE gels (that were triplicated each for statistical significance, see Figure S2) using PDQuest (BioRad, Hercules, CA, USA) 2D analysis software and compared the six gels to each other to calculate the Pearson ‘s coefficient of correlation (r) (See Figure 2).

All gels showed a strong positive correlation among them (r > 0.7), with small deviations. The correlation inside the gel group that was made with “WITA” solutions ranged from 0.81 to 0.85, and the correlation inside our “New mix” gel group varied between 0.87 and 0.9. The correlation coefficient ranged from 0.71 to 0.77 when compared between the two groups. Slightly higher correlation coefficients between our gels suggest higher reproducibility between technical replicas. The number of resolved spots was very similar—about 800 spots both in our “New mix” (av. 795 spots) and “WITA” (av. 807 spots) gels.

To further test our restored NEPHGE first-dimension gel composition, we used it for our comparative proteomics experiment to analyse the secretion of human recombinant CALR protein in *S. cerevisiae*.

### 3.2. Expression and Secretion of Human Recombinant CALR Protein in Yeast S. cerevisiae

In order to obtain samples for this comparative proteomics experiment, we expressed full-length human recombinant CALR protein precursor with its native signal sequence under an inducible *GAL7* promoter in *S. cerevisiae* strain AH22. The cells were grown in YEPD medium containing glucose for 21 h before changing the culture growth medium to YEPG containing galactose. In our previous work, we determined that *S. cerevisiae* strain AH22 reaches the diauxic shift phase—when glucose is depleted in the growth medium and cells switch from glucose fermentation to the aerobic utilisation of accumulated ethanol [34]—in 21 h of cell culture growth [19]. Because *GAL* genes can only be induced to a considerable extent at a threshold glucose concentration, introducing galactose into a cell culture during the diauxic shift, when glucose is completely depleted, results in faster GAL regulon activation [35] and swift induction of *CALR* gene under *GAL7* promoter.

According to the data collected from densitometric analysis of SDS-PAGE gels—18 h after the induction of recombinant protein expression, the secretion yield of the mature CALR was approximately 138 mg from 1 L (in the range from 120–160 mg/L) of yeast culture medium (See Figure S3A). To assess the growth phase, when the secretion of CALR was most efficient, we performed densitometric evaluation of SDS-PAGE gels loaded with yeast culture medium samples taken every three hours from the start of the recombinant protein induction (See Figure S3B). We measured relative volumes of each protein band and calculated that the biggest increase in CALR amount in the medium happened between 0 and 3 h and between 3 and 6 h (~2.8-fold) after the induction. We assumed that, if the secretion was the most effective at these growth points, the cellular proteins responsible for facilitating CALR secretion should also be more abundant. Three and six hours after the induction of recombinant protein expression were the cell culture growth points, at which we chose to take our samples for 2DE.

### 3.3. Comparative 2DE-Based Analysis of CALR-Expressing Yeast Cell Samples vs. Control

**Figure 3:**
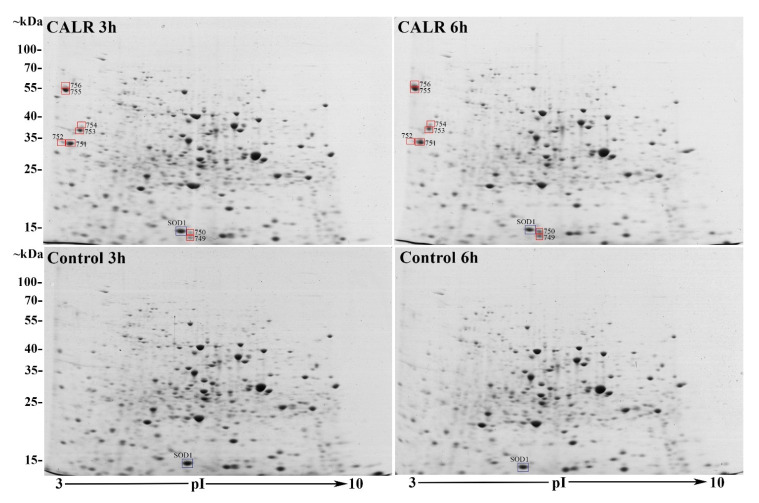
NEPHGE-based 2DE gels of CALR secreting and control yeast cell lysates at 3- and 6-h growth points after the induction with galactose, when the secretion of CALR is most efficient. All gels were loaded with 200 μg of yeast *S. cerevisiae* strain AH22 cell lysates expressing CALR protein or transformed with an empty control vector (control). Squared in red protein spots 749–756 were singled out as the most reliably differentially expressed between CALR expressing yeast samples and control. Protein spots 749 and 750 were identified as SOD1, 755–756 as CALR and spots 751–754 were identified as degradation products of CALR. Squared in blue is also a SOD1 protein spot but expressed similarly throughout all the samples.

When comparing 2DE gels of CALR-expressing cell samples versus control at 3- and 6-h growth points after the recombinant protein induction (loaded with 200 μg of yeast lysates of *S. cerevisiae* strain AH22 expressing CALR protein or transformed with empty control vector (see Section 2), we found altogether 811 different protein spots with 734 of them matching throughout all gels. We evaluated differentially expressed protein spots and found that 83 protein spots showed a fold change (FC) of 1.5 times—35 out of 811 showed increased expression in CALR gels and 48 out of 811 in control gels. The Student’s t-test analysis showed that only 35 of 811 spots were significantly differentially expressed with a *p*-value < 0.05 and only 8 of them with a *p*-value < 0.01 (See Figure 3). Unfortunately, most of them were on the brinks of the gels or were extremely small and indistinct—not suitable for excision and identification.

**Figure 4:**
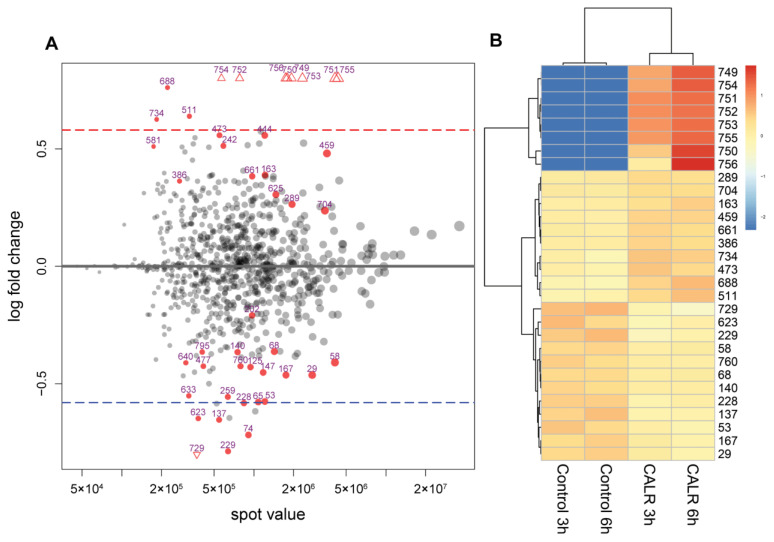
Representation of differentially expressed protein spots in a NEPHGE-based 2DE comparison of CALR-secreting and control yeast cultures. Protein spots 749–756 show the most significant differential expression. (**A**)—an MA plot (a plot of log ratio (M) and mean average (A) scales) of protein spot mean expression level (x-axis) dependence on log2FC (y-axis) after DeSeq2 analysis representing the strength of differential expression. Highly significantly differentially expressed protein spots that had highest FC and fall out of plotting area are marked as red triangles. Dashed lines represent the 0.58 log2FC thresholds: blue line marks the threshold for decreased expression and red line marks the threshold for increased expression of proteins in CALR samples. Red dots with an ID value represent proteins with with padj ≤ 0.1 value. (**B**)—a heatmap representation of 30 protein spots with the lowest *p*-value. The intensity of the colour represents how much the spot value is greater (red) or lesser (blue) than the average.

Additional DESeq2 analysis without a set FC threshold found 100 spots out of 811 to have a *p*-value ≤ 0.05 and 44 with a padj ≤ 0.1 value (padj—refers to adjusted *p*-value in DESeq2 analysis, where padj < 0.1 means that the difference is highly significant [27]). Out of 44 protein spots with highly significant differential expression (*p* ≤ 0.05), only 20 met the FC threshold of 0.58 log2FC, 11 of which showed increased expression and 9 decreased expressionin CALR samples. The highly significant spots (*p* ≤ 0.05, padj ≤ 0.1) in the CALR samples that were above FC threshold, were the most interesting to us. The 8 spots out of 11 (marked as triangles See Figure 4A) were the spots with the highest FC that fall out of the plotting area. Not surprisingly, these spots are the same ones that were identified as highly significant using the t-test (749–756) and are represented in a heatmap as having the highest spot value when calculating from the average (See Figure 4B).

The aforementioned spots with the most significant differential expression (749–756) were selected for LC-MS^E^ identification. Because of the close proximity, spots 751–752, 753–754, and 755–756 were excised as single spots. The LC-MS^E^ identified spots 751–756 as human CALR protein (accession no.: P27797, Entry CALR_HUMAN, MW 48111, pI 4.09, Calreticulin OS = *Homo sapiens*). Protein spot 755 corresponds to the mature CALR protein with the pI 4.09 and MW of ~46 kDa and spot no. 756 corresponds to the full-length un-translocated CALR precursor with intact signal peptide with MW of ~48 kDa. Spots 749–750 were identified as yeast superoxide dismutase (SOD1) (accession no.: P00445, Entry SODC_YEAST, MW 15844, pI 5.5708, superoxide dismutase [Cu-Zn] OS = *Saccharomyces cerevisiae*) (see Figure S4).

### 3.4. Comparative LC-MSE-Based Analysis of of the Whole Proteome Samples of CALR Expressing and Control Yeast Cells

2DE gel staining with CBB-R250 has the lowest protein detection limit of 8–10 ng [36]. This means that only conditionally high abundance proteins will be represented in the gel. To analyse the proteins that are less abundant, we performed a comparative whole proteome sample bottom-up tryptic analysis using LC-MS^E^. The analysis was performed using a triplicate of the 3-h after recombinant protein induction samples of CALR-secreting yeast versus control (6 samples total), to assess biological and methodological variations. In total, we identified 1726 proteins, out of which, 1574 were detected in all samples. A total of 152 proteins were not detected in a part of samples—83 were undetected in 33.3% of the samples (2 out of 6 samples) and another 69 undetected in ≥50% of samples (3 or more out of 6). Out of 1574 proteins identified in all samples, only 20 met our criteria of FC ≥1.5 times (0.58 log2FC), *p* < 0.01 and FDR < 0.05 (see Spreadsheet S2 in Supplementary Materials).

Both sample groups had underrepresented proteins. A total of 9 proteins out of 20 were not detected at all in control samples—only in CALR expressing yeast cell samples, and 4 were underrepresented in CALR samples. 60S ribosomal proteins L17-B, L26-A, L15-B, Ribose-phosphate pyrophosphokinase 2 (KPR2), Cytochrome c oxidase polypeptide 5B (COX5b), cAMP-dependent protein kinase type 3 (KAPC) and Hexose transporter HXT15 were all undetected in control samples. 3-hydroxy-3-methylglutaryl-coenzyme A reductase 2 (HMDH2) and Y’ element ATP-dependent helicase YIL177C were undetected in CALR samples. Having in mind that all the mentioned proteins are necessary for cellular housekeeping, we treated their underrepresentation in one sample and representation in another as an upregulated expression (See Table 1).

**Figure 5:**
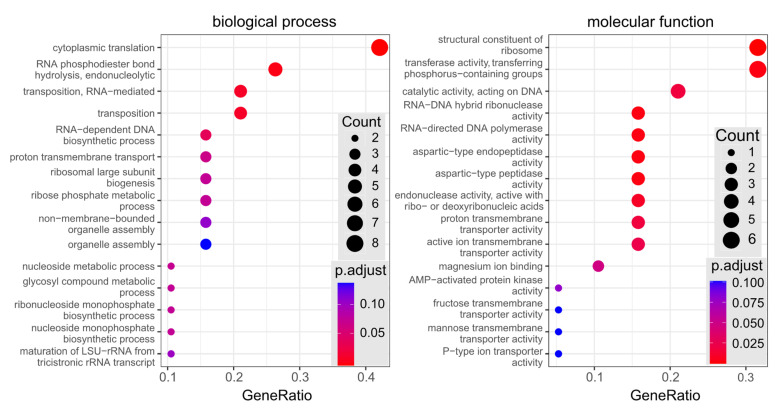
Functional enrichment analysis of differentially expressed (DE) proteins determined by LC-MS^E^ analysis. All 19 DE proteins, identified in human recombinant CALR-secreting yeast, were analysed against yeast *S. cerevisiae* genome set of GO database (see the Section 2). Dotplots depict 15 most reliably over-represented categories of two classes (biological process and molecular function) in the set of DE proteins. Dot color indicates statistical significance of category enrichment in the DE set (red—low p-adjusted value, most significant; blue—high p-adjusted value, least significant); dot size indicates number of proteins in DE set, associated with the category; horizontal axis indicates ratio: DE proteins with the category/all DE proteins.

Functional enrichment analysis of differentially expressed proteins identified by LC-MS^E^, revealed that the most overrepresented biological process in CALR-secreting samples was the cytoplasmic translation (See Figure 5: biological process). Most of differentially expressed proteins were structural constituents of the ribosome and proteins with transferase activity of phosphorus-containing groups (See Figure 5: molecular function).

## 4. Discussion

In this work, we recreated a CA composition for NEPHGE-based 2DE with the aim to analyse differential protein expression in human recombinant CALR protein-secreting yeast *S. cerevisiae*. 2DE is a robust method that allows to analyse the whole intact protein complement or proteome in high resolution, including protein isoforms and post-translationally modified proteins [70]. CA-based 2DE is a rarely used technique of 2DE, but for its capability to resolve high-protein load samples in a broad pH range and a better resolution of highly basic proteins [18], it is still used in laboratories that master this labour-intensive method. The unavailability of pre-made gel solutions may further decrease the use of this method, so we reasoned it was important to recreate the constitution of the CA in the first-dimension gels and to make it publicly available for other operators of this 2DE system. When comparing our restored recipe for NEHGE-based first-dimension separation to the commercial “WITA” gels, we got a slightly lower correlation coefficient (r = 0.71–0.77) than within the two gel groups (r = 0.87–0.9 and r = 0.81–0.85, respectively). This was to be expected, having in mind that the constitution of the CA is probably different, as the composition of “WITA” gels was never made public. A slightly better correlation between our “New mix” gels suggests better reproducibility between technical replicas. Despite the not ideal correlation between our “New mix” and “WITA” gels, they still show a strong positive correlation and our restored CA mixture and gel solutions for NEPHGE first-dimension separation can be fully used in 2DE proteomics experiments instead of the no-longer commercially available ones.

Our yeast expression system for recombinant CALR protein yielded approximately 138 mg/L of secreted protein in the culture medium. This showed about a 2-fold increase in the secreted CALR titer from our previously published results, where human recombinant CALR was expressed under yeast *PGK1* and *pFGADH1* gene promoters in *S. cerevisiae* and yielded about 60–66 mg of protein from 1 L of culture medium [17,71]. Although CALR was expressed under different promoters in this and our previous experiments, the yeast strain used, the cell culture medium, and the shake-flask growth conditions were identical, which allows us to directly compare the yield of the secreted CALR. This increase in the amount of secreted protein is facilitated by an inducible promoter vs. a constitutive promoter, and the fact that the recombinant protein expression is commenced on a higher cell culture density, allowing to maximize growth before the induction of a potentially burdening expression phase [72]. In our previous research, we also have shown that the secretion of CALR is more efficient than that of other similarly secreted ER chaperones BiP and ERp57 due to lower intracellular retention facilitated by intrinsic properties of the protein [17,73,74]. The other reason that could facilitate such an effective secretion of CALR in yeast is low free Ca^2+^ concentrations in the yeast ER lumen. It has been shown that in humans, ER calcium depletion induces CALR secretion [75], and there is 10–100 times less free Ca^2+^ in the yeast ER lumen [76] compared to the ER in human cells. High secretion titer also vastly depends on the stability of the protein. Our recent study, where the secretion titer, stability and other parameters of 50 of CALR protein mutants were analysed in yeast *S. cerevisiae*, showed that single point mutants that exhibited lower secretion titers also showed decreased protein stability [71]. In this vein, our achieved efficient 138 mg/L secretion titer of CALR asserts that the recombinant protein is highly stable and properly folded.

Considering our comparative 2DE-based analysis of CALR expressing yeast cell samples vs. the control, using t-test and DESeq2 analysis of the protein quantities in the gels, we found altogether only 8 spots that met our criteria of FC ≥ 1.5 times (*p*-value < 0.01; padj < 0.1). Although 2DE-based analysis represents only a small part of the proteome analysed, it mostly represents high abundancy proteins, which in turn can show a high degree of change in proteomes and are the most interesting [77]. It also provides us with a visual representation of the analysed proteomes—an easy way to determine just how much the cell is affected by the expression of a recombinant protein. In our case, the comparative 2DE analysis showed a very low degree of change in high abundant protein expression between samples. The overall spot patterns and intensities varied very little between CALR secreting yeast and control, with the exception of the 8 spots mentioned (See Figure 1 and Figure 2). Interestingly, in Figure 3, we distinguished a spot (blue square) that was identified in control samples as well as CALR samples as SOD1. Although this protein does not show differential expression, we identified it due to close proximity to the spots 749–750. In CALR samples, spots 749–750 appear next to SOD1 and are completely absent in control samples. It is possible that these spots are isoforms of the main cytosolic SOD1 protein. How the expression of CALR is related to the appearance of SOD1 isoforms, we have yet to find out. The secretion of CALR did not diminish in ΔSOD1 knock-out strain versus the BY4741 mother strain for control, which indicates that SOD1 is not essential for the secretion of CALR (See Figure S4) in yeast. Yet, the fact that the main SOD1 spot does not show any decrease with the appearance of the isoforms may mean that the overall expression of SOD1 is increased. Heterologous protein overexpression in yeast generates large amounts of reactive oxygen species (ROS) and induces cellular oxidative stress [78]. This happens either by the assistance of chaperones in overexpressed protein folding and disulphide bond formation [79] or by mitochondria producing large quantities of ATP for the whole process [80].

Spots 755–756 represent full-length CALR, and spots identified as CALR, but with different MW and pI positions in the gels are degradation products of the protein (spots 751–754). We identified the peptides from excised CALR protein spots, and they covered the CALR protein sequence either at the N-terminus, C-terminus or the middle (see Spreadsheet S3 in Supplementary Materials), which can only happen in the case of degradation. This suggests that despite the effective secretion of full-length CALR, there is degradation happening inside the cell. There are a few degradation pathways a protein can be sorted to undergo: ER-associated degradation (ERAD) is a process that retro-translocates mutated or improperly folded proteins from ER to the cytosol [81]; post-Golgi sorting to vacuoles [82] or endocytosis when vesicles formed at the plasma membrane enter late endosomes and then are carried to the vacuoles [83]. The amount of intact and truncated CALR protein inside the yeast cell seems to be very similar, and this suggests that the degradation is notable (See Figure 3.). The truncated parts are not secreted and must be digested in the cell. We do not yet know which degradation pathway part of CALR undergoes, but the efficient secretion indicates that it does not afflict the cell.

Our comparative whole-protein sample bottom-up tryptic analysis using LC-MS^E^ identified 1574 unique proteins. The count of identified proteins makes up a third of the total haploid yeast proteome [46,84]. Another 152 proteins were identified only in ≤50% of samples due to low protein abundance and (or) complex peptide samples. If the NEPHGE-based 2DE analysis was limited to the most abundant cellular proteins, the LC-MS^E^ analysis allowed us to examine a large part of CALR-expressing and control yeast cell proteomes.

Surprisingly, out of all the proteins identified by LC-MS^E^ and quantified using the TOP3-approach, only 20 were significantly differentially expressed (FC 1.5-fold (0.58 log2FC); *p* < 0.01; FDR < 0.05) (See Table 1). For the most part, proteins that were differentially expressed between CALR samples and the control were responsible for protein translation, energy, nucleotide, amino acid and lipid metabolism. A major group of differentially expressed proteins were structural constituents of the ribosome, according to GO functional enrichment analysis and mainly related to protein translation (See Figure S5). We did not find any differential expression in proteins involved in central carbon metabolism, protein folding or protein trafficking—which accounted for a large portion of differentially expressed proteins in similar experiments, where intracellular proteomes of yeast *Pichia pastoris* overexpressing xylanase A [85] and *Schizosaccharomyces pombe* producing α-glucosidase maltase [86] were analysed. Together with the difference in protein functional profiles, the number of proteins with changes in expression was much greater in both of the aforementioned experiments. Analysis of the proteome of *Schizosaccharomyces pombe* producing α-glucosidase maltase detected 30–40 differentially expressed proteins. Analysis of the proteome of *Pichia pastoris* overexpressing xylanase A resulted in hundreds of proteins with changes in expression and all the markers of the unfolded protein response (UPR). The relatively small number of differentially expressed proteins found in this work only confirms that CALR expression and secretion in yeast does not induce a high-level change in the proteome.

When the yeast culture medium is switched from glucose to galactose, a process impacting the whole cell metabolism, called carbon catabolite derepression (CCR) starts. Because our CALR-expressing, as well as control cells harbouring an empty vector, were both submitted to CCR, the differential expression of cellular proteins must be connected to the expression of recombinant CALR. Elevated transcription and translation rates in heterologous protein-expressing yeast are associated with increased consumption of precursors, nutrient starvation, energy and utilization of cellular machinery, which is maintained by upregulation of relevant cellular processes [87,88,89,90], and this can be recognised by looking at the functional profiles of the proteins we identified. Differential expression of eIF4G2 and ribosomal proteins may be connected to elevated translation rates in CALR-expressing samples. Increased expression of CcO5b may be induced by an elevated consumption of energy in CALR samples and reduced HMG2 levels could be the result of high flux through the sterol pathway in CALR samples due to active vesicular transport to the outer membrane. Nutrient starvation can be connected to the increased expression of HXT15. Reduced PMA2 amounts may be related to a higher protein turnover in actively secreting yeast [88].

The differential expression of PRS2 and PRS4 isoforms are hard to explain, but they do belong to different classes of ribose-phosphate pyrophosphokinases which are essential for nucleotide metabolism. It may well be that the expression of different classes of PRSs varies in time or is dependent on the nutrient starvation, which could be higher in CALR samples due to active secretion. The functions of other identified proteins which were differentially expressed between our samples are briefly presented in Table 1. While some of them can be the remnants of the recent transition to diauxic shift (Tpk3), we cannot suggest any possible connection with the secretion of CALR for others (Transposon proteins, Y’ element ATP-dependent helicase).

Many proteins participate in various biological pathways and have several functions. Merely the presence of some functions, associated with differentially expressed proteins, does not mean that these cellular functions or biological pathways are specifically affected in the test sample. To find which molecular functions and biological processes are statistically significantly overrepresented in differentially expressed proteins in comparison to all *S. cerevisiae* proteome, we performed functional enrichment analysis (Figure 5). The analysis revealed that the most significantly overrepresented biological process was associated with protein translation and the most differentially expressed proteins were structural constituents of the ribosome and proteins with transferase activity of phosphorus-containing groups. Our identified differentially expressed proteins did not have any functions related to the cellular stress or the secretory machinery. This bioinformatic analysis highlights the ribosomal proteins (RL17B, RL26A, RL15B, RL7A, RSSA1, RL22B) and the ribose phosphate pyrophosphokinases 2 and 4 from our identified protein list (See Table 1), as the members of the most statistically significantly affected functional groups. It is evident that the significant differential expression of these proteins is connected to the increased translation and nutrient requirement of CALR-secreting yeast. Taking into account the high amount of the secreted recombinant protein, an involvement of cellular functions to support protein biosynthesis by the ribosomes could be anticipated. We also expected to find other considerable changes in yeast proteome related to the processing or transport of produced CALR protein to the cell surface. However, it was not the case in this study.

The most surprising fact is that we did not find any differentially expressed proteins connected to any secretory pathway. We expected that with such a high level of secretion of recombinant CALR, the machinery of at least one secretory pathway should also demonstrate upregulation. As was mentioned before, the way that CALR exits mammalian cells is still unclear. CALR exit is mainly facilitated by ER destabilization or cellular stress [4], which leads to UPR. However, we did not find any evidence of cellular stress caused by CALR secretion from yeast cells. Yeast cells that produce recombinant proteins usually experience some degree of stress—frequently connected with folding and secretion [91,92]. Inefficient translocation to the ER leads to protein precursor accumulation in the cytosol causing UPR in the cytosol (UPR-Cyto) and insoluble aggregate formation [93]. Inefficient folding or misfolding causes UPR in the ER [94], which is directly associated with protein degradation via the ER-associated protein degradation (ERAD) pathway [95] and oxidative stress [91]. Generally, cellular stress markers associated with these responses can be used to identify the type of stress the cell is undergoing. For example, during UPR-Cyto an elevated expression of cytosolic Hsp70, Hsp90, Hsp110 heat shock stress response chaperone family proteins is detected [93], and in the case of UPR, differential expression of BiP and PDI should be noticeable [92,94]. One of the functions of BiP is redirecting misfolded proteins to degradation by ERAD [96]. The fact that we did not observe changes in the expression of BiP protein suggests that there is no active UPR and the degradation of CALR is not a consequence of ERAD. Additionally, we did not find any changes in expression in other ER-resident or cytosolic components of ERAD such as Der1p, Der3p/Hrd1p, Hrd3p or Sec61p [97,98,99,100]. No growth impairment of CALR-secreting yeast culture—which usually is a sign of stress—was observed. Lack of cellular proteins in the cell culture medium samples indicates that apoptosis is also not prevalent.

Taken together, the high-level secretion of human recombinant CALR protein from yeast *S. cerevisiae* was mostly supported by the rearrangements of protein translation machinery in the ribosomes and limited changes in the expression of cellular proteins involved in energy and metabolism. The absence of further significant changes in the secretory pathway suggests that in a steady state, yeast secretory machinery is sufficient to maintain high-level secretion of human CALR. Such a feature seems to be more characteristic to an inherently secreted protein rather than an intracellular ER chaperone. Further work is needed to compare the secretion of CALR and other naturally secreted proteins, such as human serum albumin or endogenous yeast secretory proteins. Comparative proteomic studies may help to uncover the specific mechanisms for highly efficient secretion of different proteins in eukaryotic cells and explain the dual nature of CALR being both an extracellular molecule secreted outside the cell and an intracellular chaperone residing inside the ER.

## 5. Conclusions

Efficient CALR secretion does not burden the yeast secretory machinery, only slightly impacts changes in proteome and does not cause any apparent cellular stress. The main differentially expressed proteins include structural constituents of ribosome involved in protein translation.

## Figures and Tables

**Table 1:** Function, description, log-transformed FC and FDR of the significantly differentially expressed proteins identified by LC-MS^E^ in CALR and control samples.

Cellular Proteins with Upregulated Expression in CALR Samples					
Protein Name	Description	Functional Group	Log2FC	*p*-Value (<0.01)	FDR (<0.05)
**RL17B**	60S ribosomal protein	Ribosome biogenesis [37,38,39]	13.56115	2.37783 × 10^−11^	4.1 × 10^−8^
**RL26A**	60S ribosomal protein	Ribosome biogenesis [37,38,39]	12.45301	5.09354 × 10^−11^	4.39 × 10^−8^
**RL15B**	60S ribosomal protein	Ribosome biogenesis [37,38,39]	11.49495	2.22033 × 10^−10^	1.28 × 10^−7^
**KPR**	Ribose-phosphate pyrophosphokinase 2 necessary for de novo and salvage synthesis of nucleotides	Nucleotide metabolism [40,41]	13.14576	7.28579 × 10^−10^	2.51 × 10^−7^
**COX5B**	Hypoxia-induced Cytochrome c oxidase polypeptide 5B is a terminal oxidase of the mitochondrial respiratory chain	Energy [42,43,44]	11.0593	9.82351 × 10^−9^	2.82 × 10^−6^
**YC21B**	Transposon Ty2-C Gag-Pol polyprotein	Unknown function in the cellular processes [45,46,47]	14.18216	2.47468 × 10^−6^	0.000328
**YD22B**	Transposon Ty2-DR2 Gag-Pol polyprotein	Unknown function in the cellular processes [45,46,47]	14.18216	2.47468 × 10^−6^	0.000328
**KAPC**	cAMP-dependent protein kinase type 3 essential member of the Ras signalling pathway	Regulation of cell growth, stress resistance and metabolism [48,49,50,51,52,53]	12.55319	4.67411 × 10^−6^	0.000576
**HXT15**	Hexose transporter HXT15 promotes growth of non-fermentable carbon sources in case of glucose starvation	Energy/transport [54,55,56]	9.376681	7.99509 × 10^−5^	0.00862
**RL7A**	60S ribosomal protein	Ribosome biogenesis [37,38,39]	0.782346	0.000475619	0.043181
**IF4F2**	Eukaryotic initiation factor 4F subunit p130 limiting factor of translation initiation and ribosome recruitment	Translation initiation [57,58,59,60,61]	0.574644	0.000406732	0.038978
**YD11A**	Transposon Ty1-DR1 Gag polyprotein	Unknown function in the cellular processes [45,46,47]	−16.3721	5.51007 × 10^−10^	2.38 × 10^−7^
**YN12B**	Transposon Ty1-NL2 Gag-Pol polyprotein	Unknown function in the cellular processes [45,46,47]	−10.17748	6.56423 × 10^−8^	1.59 × 10^−5^
**HMDH2**	Hypoxia induced 3-hydroxy–3-methylglutaryl-coenzyme A reductase 2 a rate-limiting member in sterol biosynthesis pathway	Lipid biosynthesis [62,63,64]	−13.22143	9.92516 × 10^−8^	1.9 × 10^−5^
**YIR7**	Y′ element ATP-dependent helicase YIL177C	Telomerase-independent telomere maintenance [65]	−10.7232	3.52 × 10^−7^	6.064 × 10^−5^
**PMA2**	Plasma membrane ATPase 2 creates proton gradient for secondary nutrient transport, elevated expression during carbon starvation	Nutrient transport and pH homeostasis [66,67,68,69]	−2.233914	1.33254 × 10^−6^	0.000209
**RSSA1**	40S ribosomal protein	Ribosome biogenesis [37,38,39]	−1.852668	1.46542 × 10^−5^	0.001685
**RL22B**	60S ribosomal protein	Ribosome biogenesis [37,38,39]	−2.51894	0.000285	0.028895
**KPR4**	Ribose-phosphate pyrophosphokinase 4, necessary for de novo and salvage synthesis of nucleotides	Nucleotide metabolism [40,41]	−1.568709	0.000516	0.044519

## Data Availability

Raw LC-MSE data are available at https://massive.ucsd.edu/ProteoSAFe/dataset.jsp?task=7839799eca504221b2c414bb3713b615 (accessed on 15 January 2022).

## Supplementary Materials

The following supporting information can be downloaded at: https://www.mdpi.com/article/10.3390/cimb44050122/s1. The structure of the human CALR globular domain depicted in the Graphical Abstract was generated using structural information deposited by [101].
