# Supplementary material for: Yeast Secretes High Amounts of Human Calreticulin without Cellular Stress
Source: Curr Issues Mol Biol. 2022 Apr 19;44(5):1768–87. doi: 10.3390/cimb44050122 (PMC9164041; doi:10.3390/cimb44050122)
Supplement: Supplementary file 1 [file cimb-44-00122-s001.zip › Figure S1-S5 and Table S1.pdf]

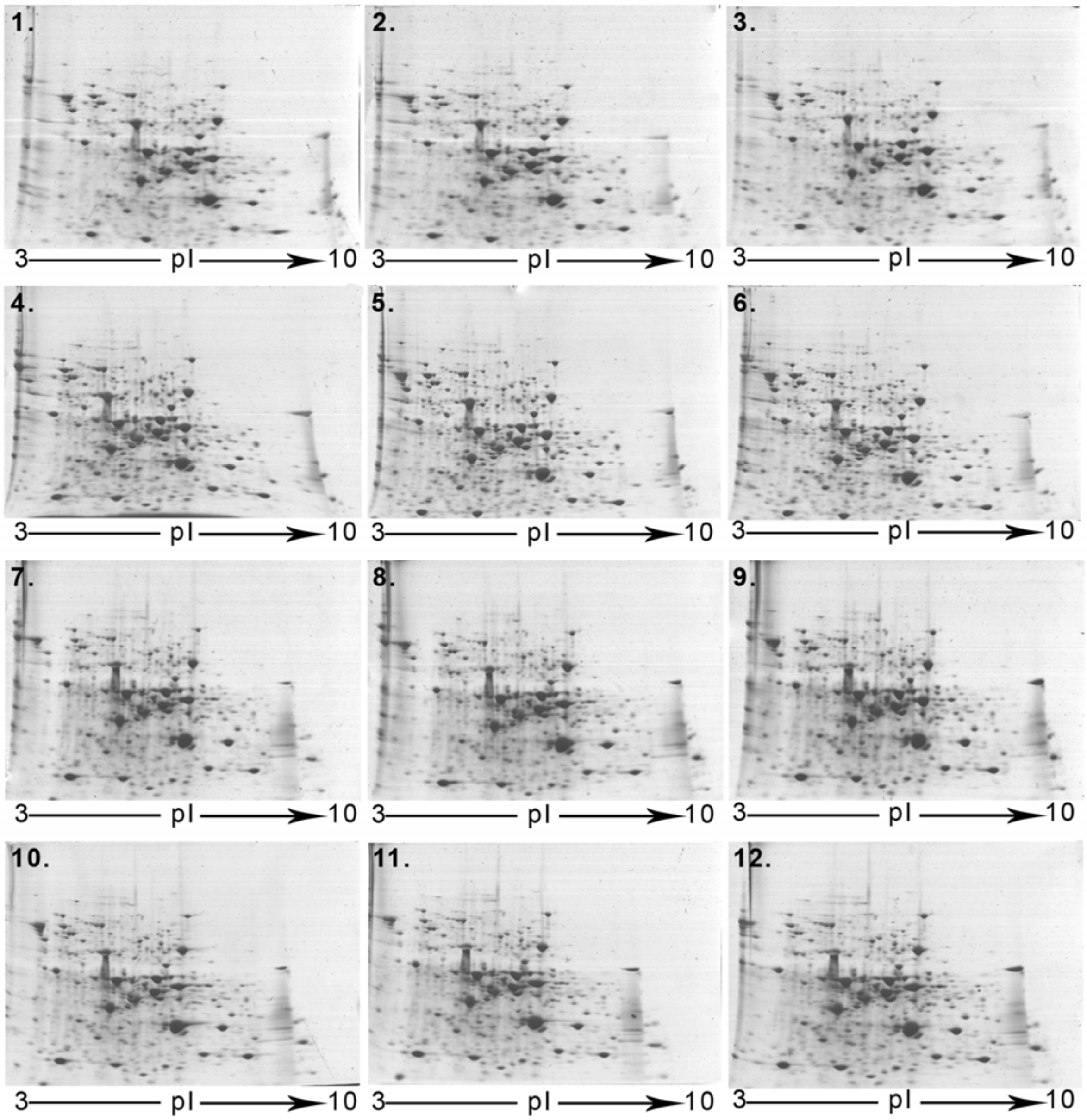

Figure S1. 2DE gels representing 12 different ampholyte mixtures used for the NEPHGE-based first-dimension separation. All gels were loaded with the same samples (80µg of lysate of *S. cerevisiae* AH22 strain cells transformed with pFGG3 vector) and the second-dimension separation was performed identically. The pI range of separated protein spots is marked on each gel. Table S1. Composition of the 12 different ampholyte mixtures used to cast NEPHGE-based first-dimension gels for 2DE.

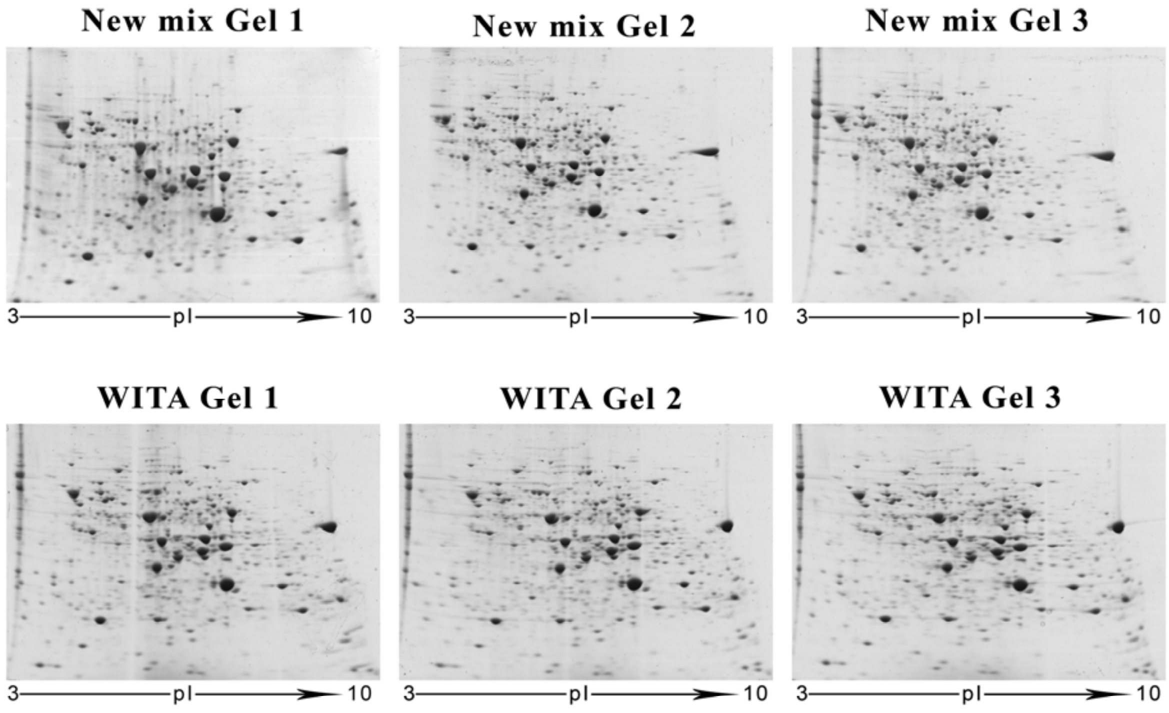

Figure S2. A triplication of 2DE separations of the same sample (80µg of lysate of *S. cerevisiae* AH22 strain cells transformed with pFGG3 vector) using either our restored "New mix" or commercial "WITA" solutions for NEPHGE-based first-dimension IEF. The pI range of separated protein spots is marked on each gel.

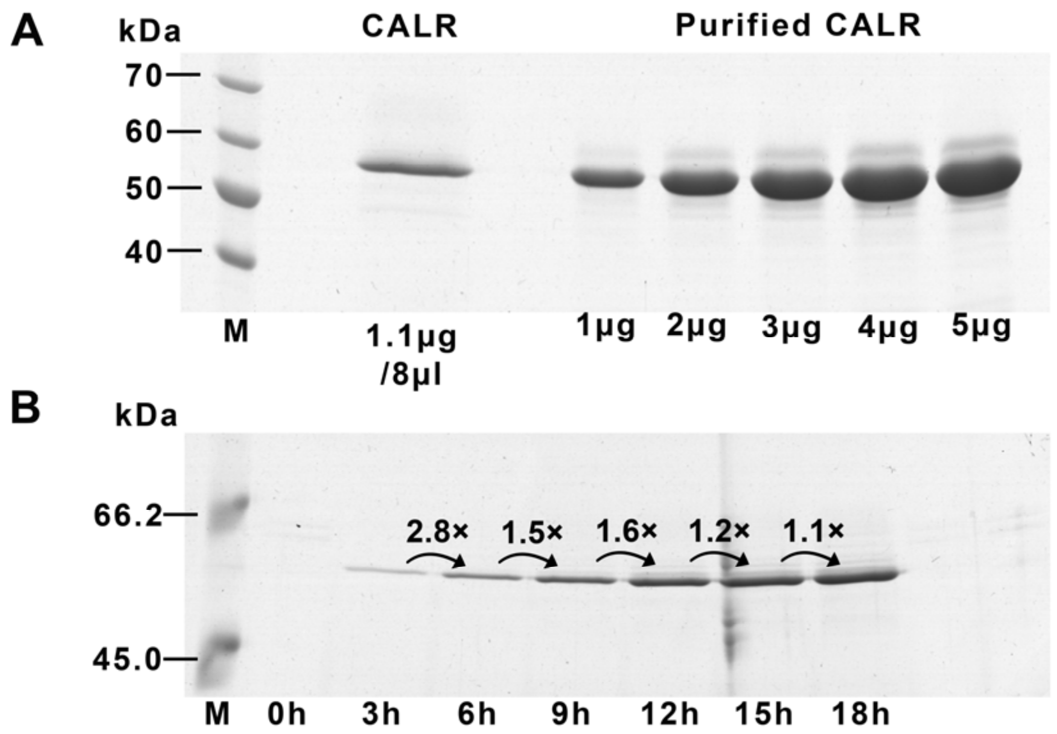

Figure S3. (**A**) – SDS-PAGE analysis of CALR-secreting yeast culture medium together with a 1-5µg of purified CALR for densitometrical quantitative calibration. 8µl of culture media containing CALR corresponds to the amount of 1.1µg, or 139 mg/L for this specific experiment. (**B**) - SDS-PAGE analysis of the secretion efficiency of CALR measured every three hours of 18-hour post-induction growth. Numbers above the arrows indicate how many times more of CALR was secreted into the culture medium between time points.

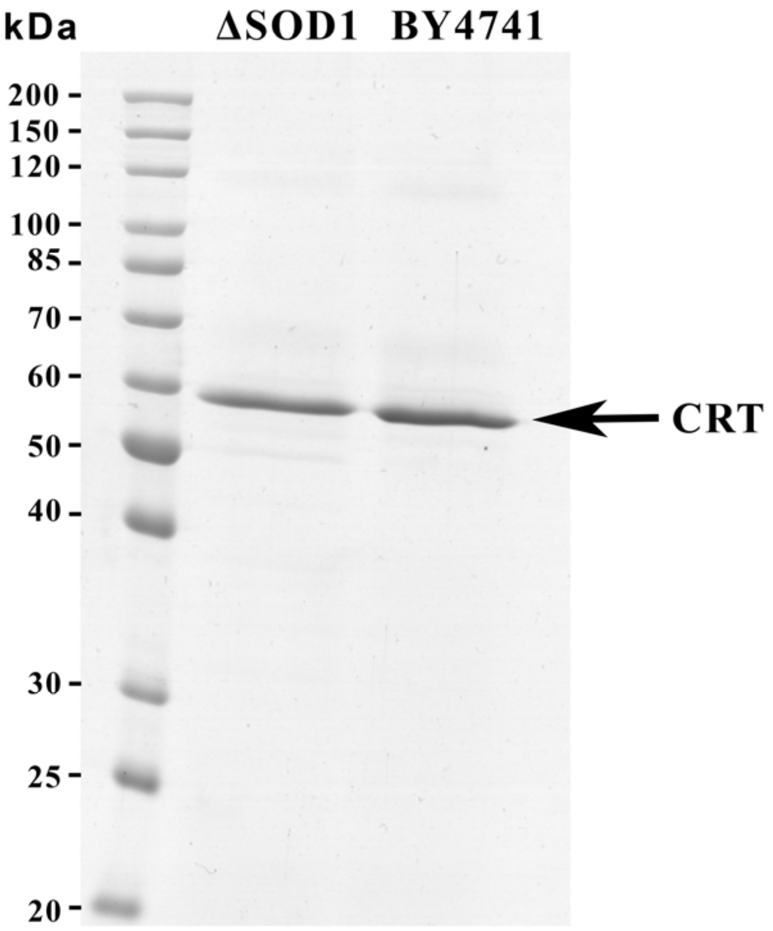

Figure S4. SDS-PAGE analysis of CALR secretion in ΔSOD1 knock-out mutant strain and its mother strain BY4741. For BY4741, 8 μl of cell culture medium was loaded. For ΔSOD1 variant the volume of cell culture medium was adjusted in accordance with cell culture optical density differences (the O.D. of ΔSOD1 culture was 1/5 times lesser at the growth end-point, that's why the volume of the loaded sample was increased 1/5 times, to normalise the CALR amount).

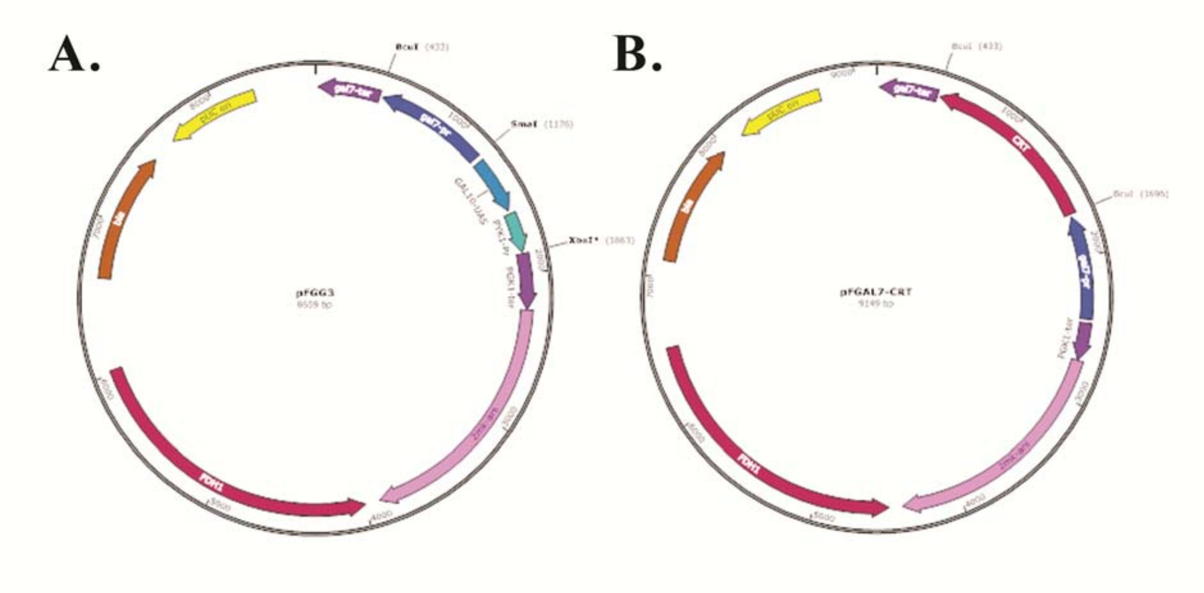

Figure S5. (**A**) – expression vector pFGG3; (**B**) – expression vector pFGAL7-CRT. 2 mkm-ars fragment of yeast 2m plasmid; bla – beta-lactamase gene conferring resistance to ampicillin; FDH1 – FDH1 gene of *Candida maltosa*, conferring resistance to formaldehyde; pUC ori – pUC origin of replication; GAL7T – *S. cerevisiae* GAL7 gene transcription terminator; GAL7P – *S. cerevisiae* GAL7 gene promoter; GAL10P – GAL10 gene UAS sequence; PYK1 – *S. cerevisiae* PYK1 gene promoter; PGK1 – *S. cerevisiae* PGK1 gene transcription terminator sequence; CRT – human Calreticulin gene.

Table S1. Composition of the 12 different ampholyte mixtures used to cast NEPHGE-based first-dimension gels for 2DE.

Mix no.	Components	Component parts in the mix	Mix no.	Components	Component parts in the mix	Mix no.	Components	Component parts in the mix
1	Servalyt<br>pH 2-11	1	2	Servalyt<br>pH 2-11	1	3	Servalyt<br>pH 2-11	1
	Pharmalyte<br>pH 5-8	2		Pharmalyte<br>pH 5-8	2		Pharmalyte<br>pH 5-8	2
	Pharmalyte<br>pH 4-6,5	3		Pharmalyte<br>pH 4-6,5	3		Pharmalyte<br>pH 4-6,5	3
	Ampholyne<br>hi-res<br>pH 6-9	1		Ampholyne<br>hi-res<br>pH 6-9	1		Ampholyne<br>hi-res<br>pH 6-9	1
	Pharmalyte<br>pH 3-10	1		Rotilyte<br>pH 3-10	1		Ampholyne<br>hi-res<br>pH 3-10	1
	Total parts:	8		Total parts:	8		Total parts:	8
4	Servalyt<br>pH 2-11	1	5	Servalyt<br>pH 2-11	1	6	Servalyt<br>pH 2-11	1
	Pharmalyte<br>pH 5-8	2		Pharmalyte<br>pH 5-8	2		Pharmalyte<br>pH 5-8	2
	Pharmalyte<br>pH 4-6,5	3		Pharmalyte<br>pH 4-6,5	3		Pharmalyte<br>pH 4-6,5	3
	Ampholyne<br>hi-res<br>pH 6-9	1		Ampholyne<br>hi-res<br>pH 6-9	1		Ampholyne<br>hi-res<br>pH 6-9	1
	Pharmalyte<br>pH 3-10	1		Rotilyte<br>pH 3-10	1		Ampholyne<br>hi-res<br>pH 3-10	1
	Pharmalyte<br>pH 8-10,5	1		Pharmalyte<br>pH 8-10,5	1		Pharmalyte<br>pH 8-10,5	1
	Total parts:	9		Total parts:	9		Total parts:	9
7	Servalyt<br>pH 2-11	1	8	Servalyt<br>pH 2-11	1	9	Servalyt<br>pH 2-11	1
	Pharmalyte<br>pH 5-8	2		Pharmalyte<br>pH 5-8	2		Pharmalyte<br>pH 5-8	2
	Pharmalyte<br>pH 4-6,5	3		Pharmalyte<br>pH 4-6,5	3		Pharmalyte<br>pH 4-6,5	3
	Rotilyte<br>pH 7-9	1		Rotilyte<br>pH 7-9	1		Rotilyte<br>pH 7-9	1
	Pharmalyte<br>pH 3-10	1		Rotilyte<br>pH 3-10	1		Ampholyne<br>hi-res<br>pH 3-10	1
	Total parts:	8		Total parts:	8		Total parts:	8

10	Servalyt pH 2-11	1	11	Servalyt pH 2-11	1	12	Servalyt pH 2-11	1
	Pharmalyte pH 5-8	2		Pharmalyte pH 5-8	2		Pharmalyte pH 5-8	2
	Pharmalyte pH 4-6,5	3		Pharmalyte pH 4-6,5	3		Pharmalyte pH 4-6,5	3
	Pharmalyte pH 8-10,5	1/3		Pharmalyte pH 8-10,5	1/3		Pharmalyte pH 8-10,5	1/3
	Rotilyte pH 7-9	1/3		Rotilyte pH 7-9	1/3		Rotilyte pH 7-9	1/3
	Ampholyne hi-res pH 6-9	1/3		Ampholyne hi-res pH 6-9	1/3		Ampholyne hi-res pH 6-9	1/3
	Pharmalyte pH 3-10	1		Rotilyte pH 3-10	1		Ampholyne hi-res pH 3-10	1
	Total parts:	8		Total parts:	8		Total parts:	8
